# Structure-function relationships in glaucoma using enhanced depth imaging optical coherence tomography-derived parameters: a cross-sectional observational study

**DOI:** 10.1186/s12886-019-1054-9

**Published:** 2019-02-15

**Authors:** Flavio S. Lopes, Igor Matsubara, Izabela Almeida, Syril K. Dorairaj, Roberto M. Vessani, Augusto Paranhos Jr, Tiago S. Prata

**Affiliations:** 1 Glaucoma Service, Department of Ophthalmology, Federal University of São Paulo, Rua Botucatu, 821, Vila Clementino, São Paulo, 04021-001 Brazil; 2 Glaucoma Unit, Hospital Medicina dos Olhos, R. Salém Bechara, 281, Centro, Osasco, 06018-180 Brazil; 3 Department of Ophthalmology, Mayo Clinic, 4500 San Pablo Rd, Jacksonville, FL 32224 USA

**Keywords:** Glaucoma, Tomography, optical, Optic nerve diseases

## Abstract

**Background:**

To investigate structural and functional correlations in glaucoma patients using enhanced depth imaging spectral-domain optical coherence tomography (EDI OCT)-derived parameters.

**Methods:**

We prospectively enrolled healthy participants and glaucomatous patients with a wide range of disease stages. All participants underwent visual field (VF) testing (SITA - Standard 24–2; Carl Zeiss Meditec, Dublin, CA) and EDI OCT imaging (Spectralis; Heidelberg Engineering Co., Heidelberg, Germany). The following optic nerve head parameters were measured on serial vertical EDI OCT B-scans by two experienced examiners masked to patients clinical data: lamina cribrosa (LC) thickness and area, prelaminar neural tissue thickness and area, anterior LC depth, Bruch’s membrane opening (BMO) and average, superior, and inferior BMO-minimum rim width (BMO-MRW). Only good quality images were considered, and whenever both eyes were eligible, one was randomly selected for analysis. Scatter plots were constructed to investigate correlations between each anatomic parameter and patient’s VF status (based on VF index [VFI] values).

**Results:**

A total of 73 eyes of 73 patients were included. All EDI OCT parameters evaluated differed significantly between glaucomatous and control eyes (*P* ≤ 0.045). A secondary analysis, in which glaucomatous patients were divided according to VF mean deviation index values into 3 groups (mild [G1; > − 6 dB], moderate [G2; − 6 to − 12 dB] and advanced [G3; <− 12 dB] glaucoma), revealed that average BMO-MRW was the EDI OCT parameter that presented more significant differences between the different stages of glaucoma. Significant structure-function correlations were found between VFI values and prelaminar neural tissue area (R^2^ = 0.20, *P* = 0.017), average BMO-MRW (R^2^ = 0.35, *P* ≤ 0.001), superior BMO-MRW (R^2^ = 0.21, *P* = 0.012), and inferior BMO-MRW (R^2^ = 0.27, *P* = 0.002). No significant correlations were found for LC area and anterior LC depth (*P* ≥ 0.452).

**Conclusions:**

Evaluating the distribution pattern and structure-function correlations of different laminar and prelaminar EDI OCT-derived parameters in glaucomatous patients, we found better results for neural tissue-based indexes (compared to LC-derived parameters). The diagnostic utility of each parameter deserves further investigations.

**Electronic supplementary material:**

The online version of this article (10.1186/s12886-019-1054-9) contains supplementary material, which is available to authorized users.

## Background

Glaucoma is a multifactorial disease, defined as a chronic, progressive neuropathy, characterized by typical changes of the optic nerve head (ONH) and retinal nerve fiber layer (RNFL), with characteristic repercussions in standard automated perimetry [1, 2]. Early diagnosis and adequate treatment continue to be the most important pillars for control and prevention of blindness due to glaucoma. Regarding glaucoma diagnosis and follow-up, functional evaluation is usually performed with visual field (VF) testing by standard automated perimetry, while structural documentation is based on retinography and optical coherence tomography (OCT) examinations [3].

When it comes to imaging tests in glaucoma, much has changed over the past few years. In this context, enhanced depth imaging spectral-domain OCT (EDI OCT) has emerged as a promising tool for evaluation of deep optic nerve structures, such as the lamina cribrosa (LC) [4–6]. As a result, new anatomic parameters and landmarks have been reported, such as Bruch’s membrane opening (BMO) and BMO-minimum rim width (BMO-MRW) [7–9].

Although the usefulness of some of these new OCT-derived parameters has been investigated (especially BMO-MRW), most of the available studies have focused solely on their diagnostic performance [7–9]. Consequently, there are scant data regarding their distribution pattern along the disease stages and structure-function correlations. Moreover, other potential EDI OCT-derived parameters, such as the prelaminar neural tissue (PLNT) area, have not been investigated in detail yet. We sought to investigate structural and functional correlations in glaucoma using laminar and prelaminar EDI OCT-derived parameters. In addition, we analyzed and compared each individual EDI OCT parameter according to disease stage.

## Methods

This observational cross-sectional study was conducted at the Department of Ophthalmology of the Federal University of São Paulo. The study protocol was approved by the Institutional Review Board of the Federal University of São Paulo and adhered to the tenets of the Declaration of Helsinki. All patients provided written informed consent prior to enrollment and examination.

### Participants

For this study, glaucomatous patients (glaucomatous optic neuropathy and reproducible VF defect) with a wide disease stage range and healthy individuals were consecutively enrolled between January 2016 and March 2017. All participants were submitted to a complete ophthalmological examination. Exclusion criteria for both groups were age younger than 18 years old, previous posterior segment intraocular surgery, ocular trauma, significant media opacity, inability to perform the examinations, and diseases affecting the eye and/or eye diseases, other than glaucoma, e. g. diabetic retinopathy, macular edema, hypertensive retinopathy.

We adopted the same glaucoma definition we used in previous studies from our group [10–12]. In brief, characteristic glaucomatous optic neuropathy was defined as a vertical cup-to-disc ratio (VCDR) of 0.6 or greater, asymmetry of VCDR of 0.2 or greater between eyes, presence of localized or diffuse peripapillary RNFL (pRNFL) defects, or neuroretinal rim defects in the absence of any other abnormalities that could explain such findings [10–12]. A glaucomatous VF defect in the standard automated perimetry (Humphrey SITA - Standard 24–2, Carl Zeiss Meditec, Dublin, CA) was defined as three or more points in clusters with a probability of less than 5% (excluding those on the edge of the field or directly above and below the blind spot) on the pattern deviation plot, a pattern standard deviation index with a probability of less than 5%, or a glaucoma hemifield test with results outside the normal limits.

As controls, we included non-glaucomatous individuals with normal VF testing and normal appearance of the optic disc, defined as a VCDR less than 0.6, without localized or diffuse pRNFL defects or neuroretinal rim thinning, and untreated intraocular pressure less than 21 mmHg [11].

### Imaging and data collection

Imaging acquisition has been thoroughly described in a previous study from our group [10]. In short, EDI OCT imaging (SD-OCT; Spectralis, Wavelength: 870 nm; Heidelberg Engineering Co., Heidelberg, Germany) was performed for both eyes of each patient and a serial of vertical B-scan images of the ONH were obtained [13, 14]. The OCT device was set to image a 15° (vertically) by 10° (horizontally) rectangle (vertical scans) centered on the optic disc with a 30° retinal window. The EDI mode of the Spectralis SD-OCT automatically positions the OCT reference plane toward the bottom of the OCT acquisition screen, shifting the zero delay to the bottom of the OCT screen, without the need of image inversion. This allows for enhanced imaging of deeper layers of ONH. This rectangle was scanned with 97 sections, with an interval between adjacent sections of approximately 30 μm, and each section had 100 OCT frames averaged.

**Fig. 1:**
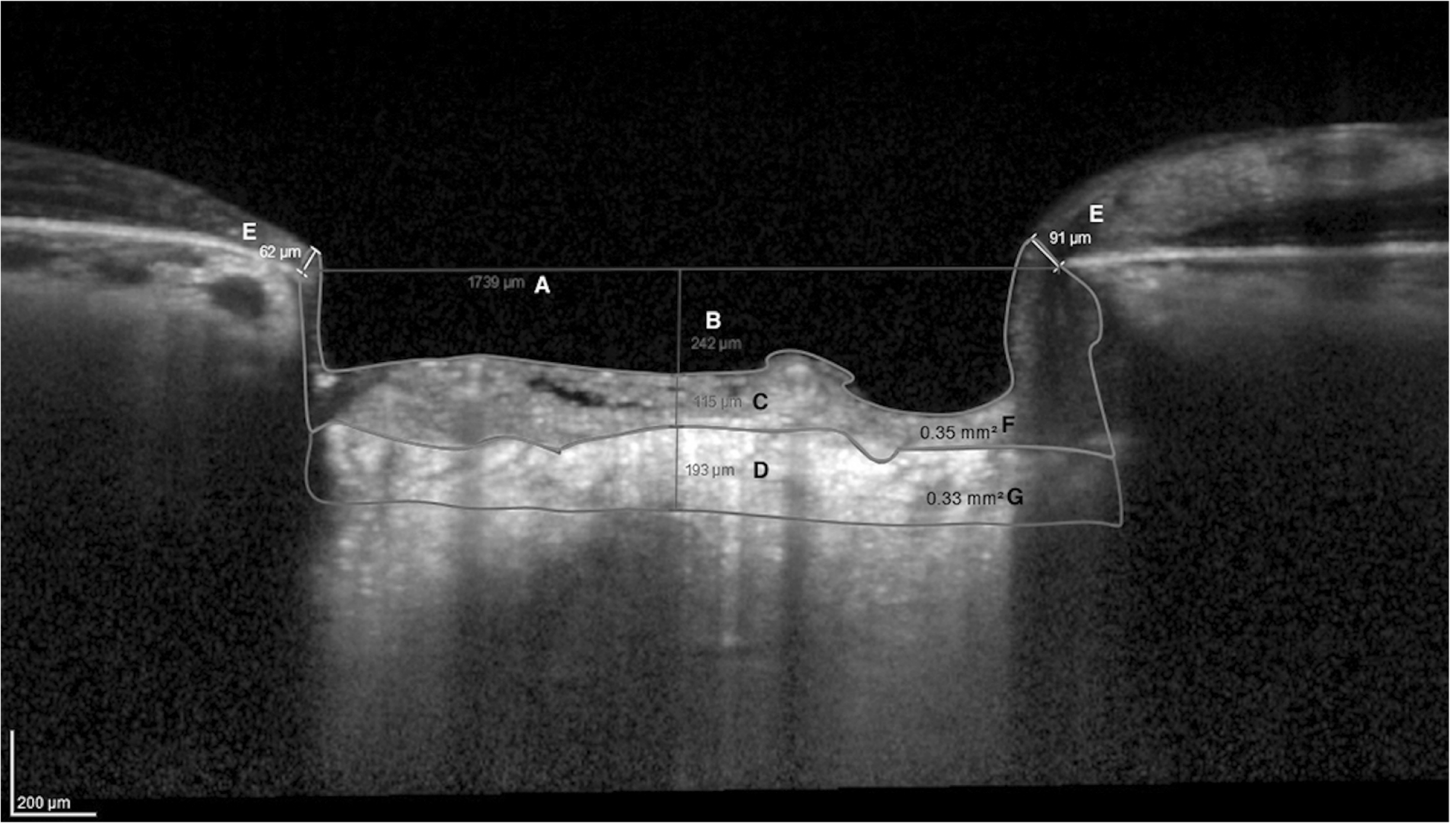
Enhanced Depth Imaging Spectral-Domain Optical Coherence Tomography B-Scan Showing Each Optic Nerve Head Parameter Evaluated. **a** Bruch’s membrane opening (BMO); **b** cup depth; **c** prelaminar neural tissue (PLNT) thickness; **d** lamina cribrosa (LC) thickness; **e** BMO-minimum rim width; **f** PLNT area; and **g** LC area

The following ONH parameters were assessed in EDI OCT vertical B-scans by experienced examiners (FSL and IA) masked to patients clinical data (Fig. 1): LC thickness and area, PLNT thickness and area, anterior LC depth (ALD), BMO, average BMO-MRW (aBMO-MRW), superior BMO-MRW (sBMO-MRW), and inferior BMO-MRW (iBMO-MRW). Only high quality images were considered for analysis, showing at least a signal-to-noise ratio greater than 25 dB as recommended by the OCT device manufacturer. To increase LC visibility at the EDI OCT scans, image contrast was optimized to the maximum level and image colors were switched (black or white) as needed using the device software. The line connecting Bruch’s membrane edges was used as a reference plane for all depth measurements [15]. The selected parameters were measured in the B-scans based on the horizontal center of the ONH using the built-in software calipers (linear measurements; unit: μm). Area measurements were also taking (manually measured) using the built-in software calipers (unit of measurement: mm^2^). If the selected image presented vascular shadows which compromised the LC or prelaminar tissues visualization, we used the closest temporal B-scan that did not present such artifacts (approximately 30 μm apart). As a result, LC and PLNT were measured as close to the horizontal center of the ONH as possible. The thickness of the LC was defined as the distance between the anterior and posterior borders of the highly reflective region at the vertical center of the ONH in the vertical EDI OCT cross-sectional B-scans [16]. PLNT was defined as the perpendicular distance between the anterior PLNT surface and the anterior surface of the LC. Cup depth was defined as the perpendicular distance from the reference line to the anterior PLNT surface. BMO-MRW was defined as the minimum distance from the inner opening of the BMO to the internal limiting membrane. PLNT area is an area of soft tissue located between the optic cup surface and the anterior LC surface [17]. It was defined as the area comprised between the sBMO-MRW and iBMO-MRW and the anterior portion of the LC (Fig. 1) [18–20]. We used the average of two measurements for each parameter. When both eyes were eligible, one was randomly selected for analysis.

### Statistical analysis

Descriptive analysis was used to present demographic and clinical data. D’Agostino-Pearson’s test was performed to determine whether data had a normal distribution. Descriptive statistics included mean and SD for normally distributed variables and median, quartiles for those non-normally distributed. Independent samples *t* test was used to compare continuous normally distributed variables between groups, while the Mann-Whitney test was used to compare those non-normally distributed. Categorical data were compared using χ^2^ test.

We also analyzed each individual EDI OCT parameter by disease stage. Patients were divided according to VF mean deviation (VFMD) index values into 3 groups: mild (G1), moderate (G2), and advanced (G3) glaucoma (> − 6 dB, − 6 to − 12 dB, and < − 12 dB, respectively). Scatter plots were constructed and regression analysis was used to investigate the correlations between each anatomic parameter and patient’s VF status (based on VFMD index and VF index [VFI] values).

#### Sample size calculation

The ONH parameter chosen for sample size calculation was PLNT thickness, which was compared between glaucomatous and control eyes in our study (among other parameters). Previously reported PLNT thickness values are approximately 300 μm in healthy eyes [21]. Considering a mean expected difference of 30% (between study and control eyes) and an estimated mean standard deviation in each sample of 50%, for an α error of 0.05 a minimum of 66 patients (33 patients in each group based on a 1,1 ratio) would be necessary to reach a statistical power of 80%. Computerized analysis was performed using MedCalc software (MedCalc Inc., Mariakerke, Belgium). The alpha level (type I error) was set at 0.05.

## Results

A total of 73 eyes from 73 participants (39 patients with glaucoma and 34 controls) were enrolled. Another 8 eyes of 8 patients were not included in the analysis due to poor quality images or inability to define the posterior limits of the LC. Among included patients, all data were available for analysis at the end of the study. Table 1 provides clinical and ocular characteristics of included patients. Age, race, and axial length were similar between the two groups (*P* ≥ 0.122). The proportion of women in the glaucoma group (33%) was lower than the control group (65%; *P* = 0.014). Mean intraocular pressure values were similar between groups (*P* = 0.882). Most glaucoma patients had primary open-angle glaucoma (69%). Other diagnoses included primary angle-closure glaucoma (20%), secondary glaucoma (8%) and pigmentary glaucoma (3%). Glaucoma patients were using 1.6 ± 1.1 medications on average). As expected, VFI, VFMD, and average pRNFL thickness differed significantly between patients and controls (*P* < 0.01; Table 1).

**Table 1:** Demographic and Ocular Characteristics of Study Patients

Variables^a^	Control Group (*n* = 34)	Glaucoma Group (*n* = 39)	*P* Value
Age, y	60.7 ± 10.7	64.9 ± 11.9	.122
Sex, No. female/male	22/12	13/26	.014
Race, % W/AD/O	52/11/37	47/25/28	.582
IOP, mm Hg	14.2 ± 2.5	14.1 ± 3.2	.882
CCT, μm	547 ± 31	511 ± 38.1	<.01
Axial length, mm	23.1 ± 0.9	23.1 ± 0.8	.794
Average pRNFL thickness, μm	101.1 ± 14.3	65.2 ± 12.4	<.01
VFMD, dB	-1 ± 0.9	−9.4 ± 7.4	<.01
VFI, %, median (Q1, Q3)	99 (98, 100)	83 (62.2, 91.2)	<.01

Abbreviations: *AD* African descendants, *CCT* Central corneal thickness, *IOP* Intraocular pressure, *O* Other, *pRNFL* Peripapillary retinal nerve fiber layer; *Q1* First quartile, *Q3* Third quartile, *VFI* Visual field index, *VFMD* Visual field mean deviation, *W* white

^a^Data are given as mean ± SD unless otherwise indicated

**Fig. 2:**
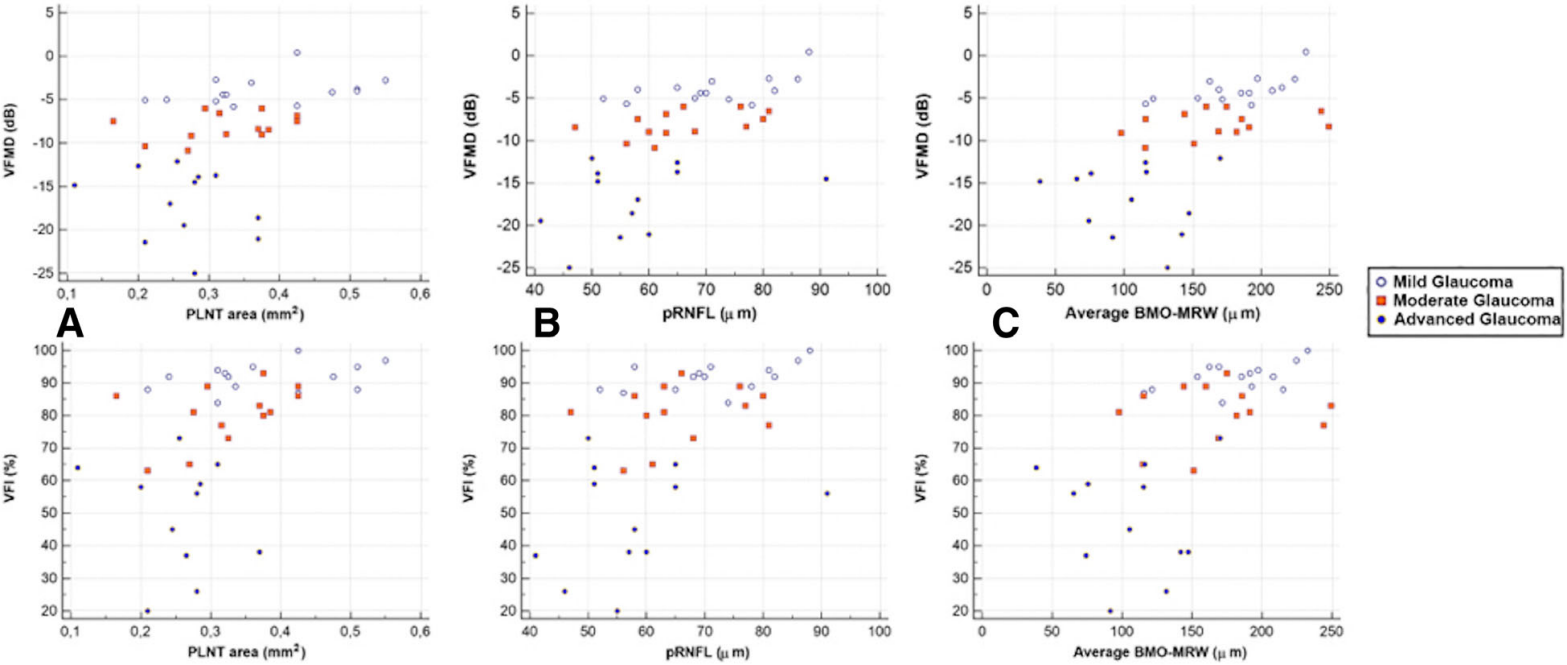
Scatter plots depicting the association between global visual field indices (VFMD and VFI) and different EDI OCT parameters (PLNT Area, pRNFL Thickness and Average BMO-MRW). BMO-MRW indicates Bruch’s membrane opening-minimum rim width; PLNT, prelaminar neural tissue; pRNFL, peripapillary retinal nerve fiber layer; VFMD, visual field mean deviation; and VFI, visual field index

When it comes to EDI OCT-derived ONH, all parameters evaluated differed significantly between glaucomatous eyes and controls (*P* ≤ 0.045; Table 2). Regarding the structure-function correlations we investigated in our glaucoma population (control eyes were not included in the regression analysis), the following parameters were significantly associated with VFMD values: PLNT area (R^2^ = 0.20, *P* = 0.0043), pRNFL thickness (R^2^ = 0.32, *P* = 0.0002), aBMO-MRW (R^2^ = 0.38, *P* ≤ 0.0001), sBMO-MRW (R^2^ = 0.27, *P* = 0.006) and iBMO-MRW (R^2^ = 0.27, *P* = .0006). Compared with VFI values: PLNT area (R^2^ = 0.20, *P* = 0.017), pRNFL thickness (R^2^ = 0.32, *P* ≤ 0.001), aBMO-MRW (R^2^ = 0.35, *P* ≤ 0.001), sBMO-MRW (R^2^ = 0.21, *P* = 0.012) and iBMO-MRW (R^2^ = 0.27, *P* = 0.002). No significant correlations were found for LC thickness or area and ALD when compared with both VFMD and VFI (*P* ≥ 0.452). The structure-function correlations between VFMD and VFI values and PLNT area, pRNFL thickness and aBMO-MRW are shown in Fig. 2a, b, and c, respectively. Overall, we documented non-linear patterns. We believe that these non linear relationships between structure and function loss could be in part explained by the well described ceiling effect of the VFI [22, 23] which tends to present high values in the early stages of the disease despite significant structural loss. In addition, such findings could be related to the intrinsic behavior of the structure-functional loss in glaucoma itself. Usually, there is a more rapid axonal loss before visual function deterioration is documented, especially in the early stages of the disease [24–27].

**Table 2:** Comparison of Optic Nerve Head Parameters between Glaucomatous and Control Eyes

Parameters^a^	Control Group (*n* = 34)	Glaucoma Group (*n* = 39)	*P* Value
PLNT thickness, μm	269.1 ± 173	98.3 ± 49.44	<.001
PLNT area**,** mm^2^	0.65 ± 0.17	0.33 ± 0.09	<.001
BMO, μm	1497.5 ± 175.2	1589.8 ± 208.1	.045
Cup depth, μm	144.2 ± 152.8	379.8 ± 150.5	<.001
ALD, μm	413.3 ± 109.1	478.2 ± 140.1	.032
LC thickness, μm, median (Q1, Q3)	161.5 (139.5, 188)	141.9 (37.3, 145)	.004
LC area, mm^2^	0.24 ± 0.04	0.20 ± 0.05	.001
aBMO-MRW, μm	328.6 ± 55.2	153.7 ± 50.7	<.001
sBMO-MRW, μm	330.1 ± 60.4	158 ± 61.3	<.001
iBMO-MRW, μm	327.1 ± 57.8	149.4 ± 59.3	<.001

Abbreviations: *aBMO-MRW* Average Bruch’s membrane opening-minimum rim width, *ALD* Anterior lamina depth, *BMO* Bruch’s membrane opening, *iBMO-MRW* Inferior Bruch’s membrane opening-minimum rim width, *LC* Lamina cribrosa, *PLNT* Prelaminar neural tissue, *Q1* First quartile, *Q3* Third quartile, *sBMO-MRW* Superior Bruch’s membrane opening-minimum rim width

^a^Data are given as mean ± SD unless otherwise indicated

Table 3 provides a comparison of different EDI OCT parameters between controls and glaucomatous eyes (divided according to disease stage). In addition to laminar and prelaminar EDI OCT parameters, conventional pRNFL values were also included for comparison. Significant differences between controls and at least one of the glaucomatous groups were found for all parameters (*P* < 0.001), except for ALD (*P* = 0.177). BMO-MRW was the EDI OCT parameter that presented more significant differences between the different stages of glaucoma. In general, a descriptive analysis suggested that all parameters (except ALD) seem to change gradually, from controls to the most advanced stages of the disease. When it comes to ALD changes, even though not statistically significant, eyes with mild-to-moderate glaucoma appear to have greater ALD values (deeper cups) than controls. However, in eyes with more advanced disease, mean ALD values seem to decrease, as mean values for eyes with advanced glaucoma were smaller than those for eyes with mild-to-moderate disease.

**Table 3:** Optic Nerve Head and Retinal Nerve Fiber Layer Parameters by Disease Stage

Parameters^a^	Control Group (*n* = 34)	Mild Glaucoma (*n* = 14)	Moderate Glaucoma (*n* = 13)	Advanced Glaucoma (*n* = 12)	*P* Value
PLNT area, mm^2^	0.65 ± 0.17	0.38 ± 0.1	0.32 ± 0.08	0.26 ± 0.07	<.001^b^
Average pRNFL thickness, μm	101.1 ± 14.3	71.3 ± 11.1	65.9 ± 10.2	57.5 ± 12.8	<.001^c^
ALD, μm	413.3 ± 109.1	476.4 ± 98.1	494.9 ± 162.3	462.1 ± 164.4	.177
Average BMO-MRW, μm	328.6 ± 55.2	181.5 ± 35.2	167.6 ± 45.6	106.1 ± 38.4	<.001^d^

Abbreviations: *ALD* Anterior lamina depth, *BMO-MRW* Bruch’s membrane opening-minimum rim width, *PLNT* Prelaminar neural tissue, *pRNFL* Peripapillary retinal nerve fiber layer

^a^Data are given as mean ± SD

^b^Each glaucoma group differed significantly from controls, but there was no significant difference between the glaucoma groups

^c^Each glaucoma group differed significantly from controls. There was also a significant difference between eyes with mild and advanced glaucoma

^d^Each glaucoma group differed significantly from controls. There were also significant differences between eyes with mild and advanced glaucoma, and between eyes with moderate and advanced glaucoma

These data were presented in part at the Annual Meeting of the Association for Research in Vision and Ophthalmology 2017. The complete raw data from glaucomatous and control eyes are available in Additional file 1.

## Discussion

During the last two decades, imaging advancements (especially OCT technology) have not only yielded a better evaluation and quantification of both ONH and RNFL parameters, but have also contributed significantly to a deeper comprehension of the relationship between structure and function in glaucoma. However, it has been shown that the use of conventional pRNFL analysis does not apply for every eye, either due to technical limitations or ocular characteristics, such as peripapillary atrophy, disc tilt, high myopia, or end-stage glaucoma [28–30]. As a result, researchers are gradually returning to investigate the topographic characteristics of the ONH with more attention [7, 14, 18, 19, 31]. In this context, both laminar (e.g. laminar depth, thickness, and localized defects) and prelaminar parameters (neural tissue-based metrics, such as BMO-MRW) have been recently evaluated in eyes with and without glaucoma [4–6, 8, 13, 15, 20, 21, 31–36]. In our study, evaluating structural and functional correlations of different ONH parameters using EDI OCT in eyes with different glaucoma stages, we found better results for neural tissue-based indexes (compared to LC-derived parameters). We believe that our findings not only add to the scant data on the structure-function relationships between laminar/neural tissue-based parameters and VF loss in glaucoma, but also provide new data about how some of these new OCT parameters, such as PLNT area, correlate with patients’ functional status.

Structure-function relationships in glaucoma using the conventional pRNFL thickness analysis have already been extensively investigated in the literature [24, 37–39], and thus were not the main focus of our study. Concerning the studies evaluating BMO-MRW OCT protocol, even though its diagnostic performance and test-retest variability have been well documented [8, 9, 36, 40, 41], not much has been investigated about its structure-function correlations. In general, good structure-function correlations between BMO-MRW thickness and VF sensitivity have been documented, usually revealing a non-linear pattern (similar to what we found in our study). Pollet-Villard et al. [9] reported R^2^ values ranging between 0.26 and 0.66, depending on the chosen ONH sector. In our study, R^2^ values ranged between 0.21 and 0.35. It should be noted that while Pollet-Villard et al. [9] used a sectorial ONH analysis (clock hours), we adopted a more generalized analysis (average, superior, and inferior). Concerning other neural tissue-based EDI OCT parameters, more specifically the PLNT area, we were able to find one recently published study. The authors investigated PLNT area changes following trabeculectomy and non-penetrating deep sclerectomy, but did not evaluate the parameter’s diagnostic performance nor structure-function relationships [17]. In our study, although we found a significant structure-function correlation between PLNT area and VF loss (VFI values) in glaucomatous eyes, such correlation was weaker than those we found for the conventional pRNFL thickness analysis and BMO-MRW protocol. Overall, considering the analyses we performed, we believe our findings not only corroborate previously published data on structure-function correlations in glaucoma, but also provide additional information concerning neural tissue-based EDI OCT parameters.

In our study, three laminar parameters were evaluated: LC thickness, area, and depth. Even though these parameters differed significantly between glaucomatous eyes and controls, we did not find any significant structure-functional correlation between LC-derived parameters and VFI values. Our comparative laminar findings are in agreement with most previously published data, as it has been shown that glaucomatous eyes present thinner and deeper LCs than controls [10, 16]. In addition, our structure-functional findings on LC depth corroborate those from Park et al. [31], as the authors did not find a significant association between ALD and VFMD values in eyes with different stages of treated glaucoma. Interestingly, when evaluating ALD values in healthy eyes and eyes with different glaucoma stages, although we found greater ALD values in eyes with mild-to-moderate glaucoma (deeper cups than controls), the same did not occur in eyes with more advanced disease, as mean ALD values seemed to decrease in these eyes. This LC depth pattern across the disease stages could be explained in part by a recent study by Tun et al. [42]. Evaluating the correlation between VF indexes values and pressure-induced LC displacement, the authors found that while patients with mild glaucoma presented a posterior LC displacement, those with more advanced disease had an anterior LC displacement [42–44]. These data suggest that much of the LC depth changes in glaucoma patients appear to occur in the early stages of the disease.

Some specific characteristics and limitations of our study should be considered. First, our results apply solely to this specific study population, and therefore, should not be extrapolated to glaucomatous patients with different characteristics. Second, it must be emphasized that the analysis of LC changes, which was not evaluated in this study, could be a more useful parameter than the measurement of the LC position alone. Third, there is still no consensus on whether the BMO could be used as an adequate reference for LC measurements [20, 45, 46]. Finally, our correlation analyses did not take into consideration possible confounding factors, such as disc size, axial length, and age, which could be related to the main variables being investigated in the study.

## Conclusions

While assessing the distribution pattern and structure-function correlations of different laminar and prelaminar EDI OCT-derived parameters in glaucomatous patients, we found better results for neural tissue-based indexes (compared to LC-derived parameters). We understand that such parameters could add to the conventional pRNFL protocol for structural glaucoma evaluation, especially in eyes whose anatomical features limits the use of the conventional analysis. The diagnostic utility of each parameter still needs to be investigated.

## Additional file

Additional file 1 Raw Data for BMC ophthalmology. Data of controls and glaucoma groups. (XLS 58 kb)

## Abbreviations

aBMO-MRW: Average Bruch’s membrane opening-minimum rim width
BMO: Bruch’s membrane opening
BMO-MRW: Bruch’s membrane opening-minimum rim width
EDI OCT: Enhanced depth imaging spectral-domain optical coherence tomography
iBMO-MRW: Inferior Bruch’s membrane opening-minimum rim width
LC: Lamina cribrosa
OCT: Optical coherence tomography
ONH: Optic nerve head
PLNT: Prelaminar neural tissue
pRNFL: Peripapillary retinal nerve fiber layer
RNFL: Retinal nerve fiber layer
sBMO-MRW: Superior Bruch’s membrane opening-minimum rim width
VCDR: Vertical cup-to-disc ratio
VF: Visual field
VFI: Visual field index
VFMD: Visual field mean deviation
